# A rare complication of pulmonary tuberculosis: a case report

**DOI:** 10.1186/s13104-015-0990-6

**Published:** 2015-02-10

**Authors:** Kulatunga Wijekoon Mudiyanselage Pramitha Prabhashini Kumarihamy, Dissanayake Mudiyanselage Priyantha Udaya Kumara Ralapanawa, Widana Arachchilage Thilak Ananda Jayalath

**Affiliations:** University Medical Unit, Teaching Hospital, Peradeniya, Sri Lanka; Department of Medicine, University of Peradeniya, Peradeniya, Sri Lanka

**Keywords:** Pulmonary tuberculosis, Deep vein thrombosis, Pulmonary embolism

## Abstract

**Background:**

Pulmonary tuberculosis remains an important public health problem globally and one of the most prevalent infectious diseases in Sri Lanka. It can cause a wide variety of complications but hematological manifestations are rare. According to our literature survey, this is the first reported case of the disease associated with deep vein thrombosis in Sri Lanka.

**Case presentation:**

A 37 year old Sri Lankan Sinhalese female presented with fever of one month’s duration with productive cough and two weeks painless left lower limb swelling. Chest X-ray showed bilateral inflammatory shadows with a cavitatory lesion on the right apical region. A computed tomographic pulmonary angiography scan excluded pulmonary embolism. She had rising mycoplasma antibody titre (four fold). Acute deep vein thrombosis of the left lower limb was confirmed by venous duplex. Pulmonary tuberculosis was confirmed with positive culture for Mycobacterium tuberculosis. She was treated with clarythromycin, enoxaparin, warfarin and anti tuberculus drugs. It was difficult to maintain her International Normalizing Ratio in the therapeutic range due to drug interactions and poor compliance. At five months of presentation she died of massive pulmonary embolism.

**Conclusion:**

Our case emphasizes that patients with severe pulmonary tuberculosis are at risk of developing thromboembolism and superadded infections. It should be noted that even though starting anti tuberculosis drugs improved haemostatic disturbances, achieving the target International Normalizing Ratio was difficult due to drug interactions. Therefore these patients should be closely followed up to prevent complications and death from pulmonary embolism.

**Electronic supplementary material:**

The online version of this article (doi:10.1186/s13104-015-0990-6) contains supplementary material, which is available to authorized users.

## Background

According to the WHO (World Health Organization), every second a person gets infected by the tuberculus bacillus in the world. As of today one third of world's population has been infected by this bacillus. Tuberculosis is the commonest cause for death in adults with infectious diseases despite the availability of drugs. Therefore it is a health problem globally. Tuberculosis can cause a wide variety of complications but hematological complications are rarely demonstrated in pulmonary tuberculosis. Some authors believe that the risk of developing deep vein thrombosis (DVT) increases with the severity of this disease [1].

DVT can be the presenting feature of tuberculosis that occurs late in the course of this disease or in patients on anti tuberculosis treatment [2-4]. Here we present a case of pulmonary tuberculosis presented to us with DVT in the lower limb. Even though tuberculosis is one of most prevalent infectious disease in Sri Lanka and to the best of our knowledge after through literature searched, this is to be the first reported case of pulmonary tuberculosis (PTB) presenting with DVT.

## Case presentation

A 37 year old Sri Lankan Sinhalese female presented to the Teaching Hospital Peradeniya, Sri Lanka with fever of one month duration. She complaints of productive cough of three week’s duration with no history of haemoptysis or breathlessness. Fever was associated with anorexia and weight loss. She noticed painless left lower limb swelling of two weeks duration which had gradually extended up to the upper thigh three days before presenting to the hospital. She did not have any history of joint pain, photosensitivity rash or alopecia, deep vein thrombosis, recurrent abortion, intrauterine death or thrombotic stroke. She did not have risky behavior or history of immobilization. She was neither an alcoholic nor smoker and denied use of oral contraceptives.

On examination she was febrile and pale but not tachypnic or tachycardic. She was normotensive and the pulse oxymetric reading was 96% on air. Chest auscultation revealed scattered bilateral coarse crepitations which were more on the right side. Abdominal examination was normal including per vaginal and per rectal examination. Cardiovascular and neurological examinations were clinically normal. Her left lower limb was swollen up to the groin and there was tenderness over the calf. No evidence of cellulites was detected. On the third day of hospital admission she became dyspnoic with wide spread right sided coarse crepitations and reduced pulse oximetric reading of 93% on air.

**Figure 1:**
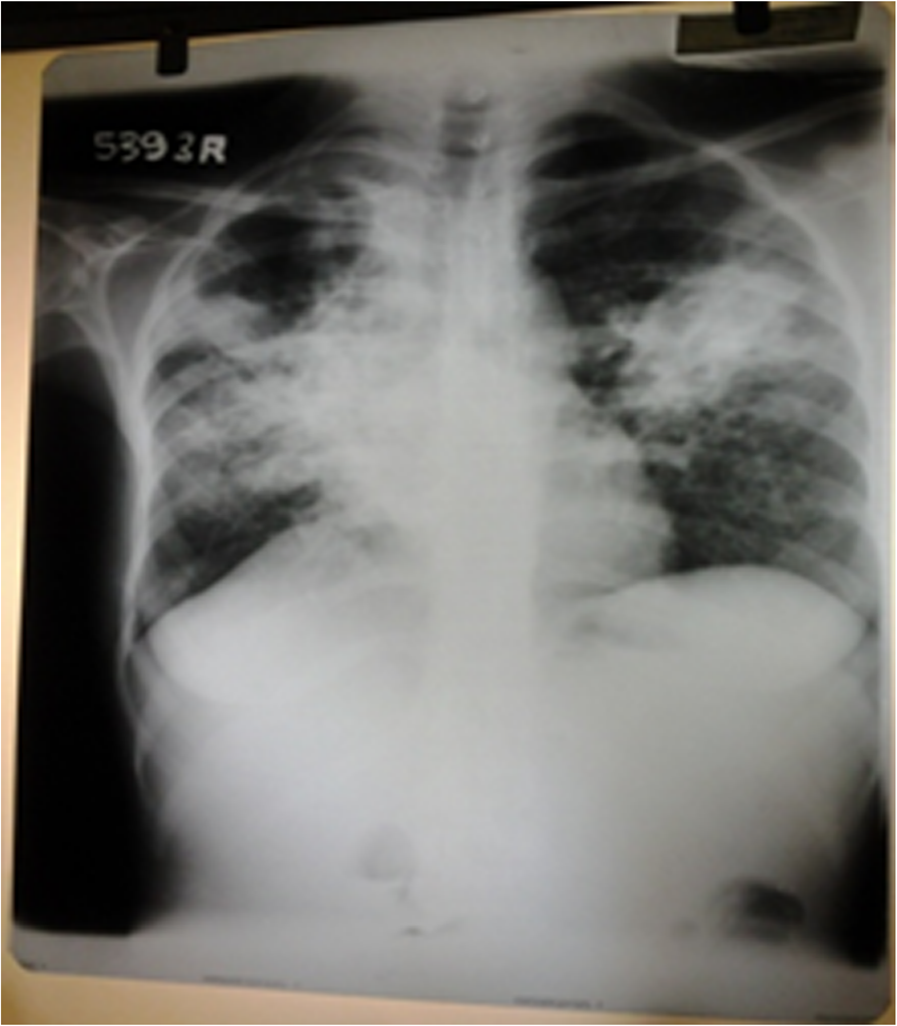
**(Chest x-ray on Day 1 of admission).** Opacification predominantly involving the right mid zones. There are adjacent nodular opacities with large cavitatory lesion at the right upper zone. Right heart border is obliterated. Mediastinum has shifted to right. No pleural effusions or bony erosions.

**Figure 2:**
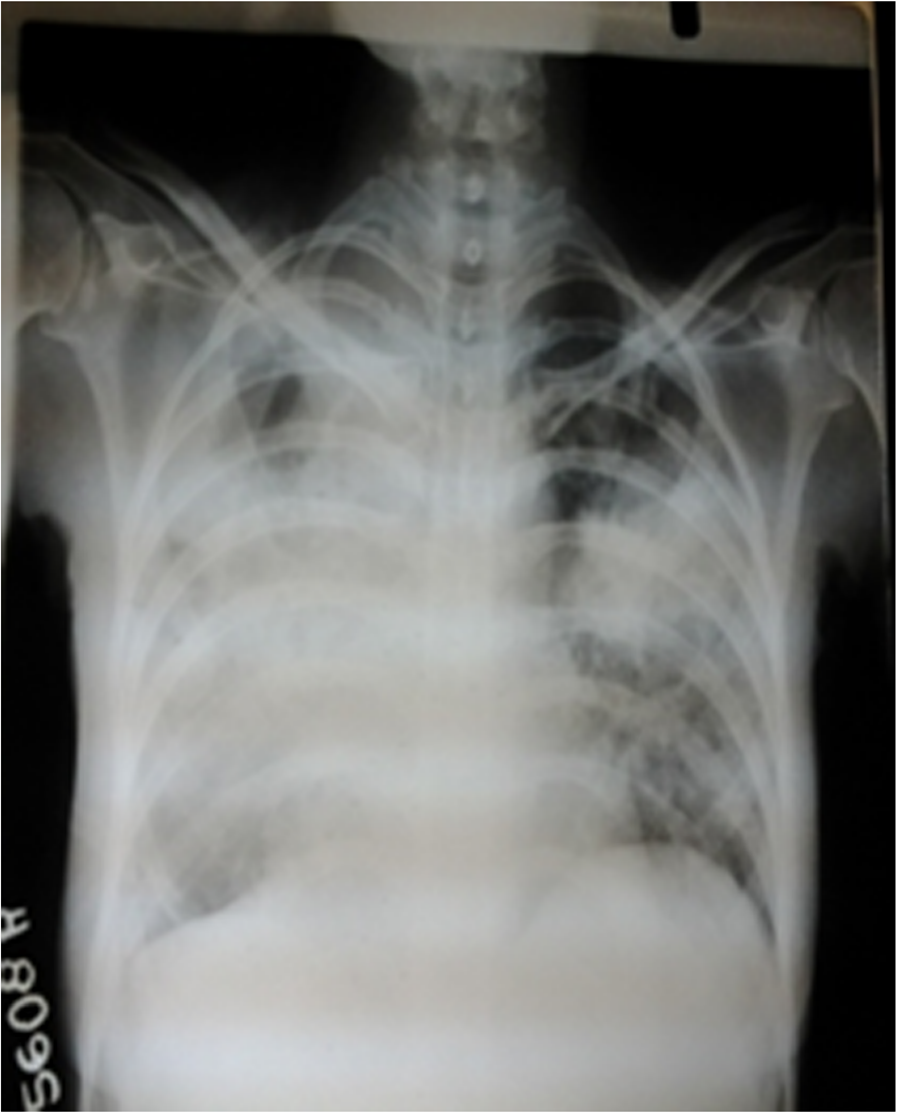
**(Chest x-ray on Day 3 of admission).** Extent of opacification increased with coalescing of nodules (Right > Left).

Laboratory finding on admission revealed relative neutrophil leucocytosis, hypochromic microcytic anaemia (haemoglobin 10.5 g %) with high Erythrocyte sedimentation rate (ESR) and C-reactive protein (CRP). The blood picture revealed, hypochromic microcytic anaemia with toxic granules and vacuolations in neutrophils indicating severe bacterial infection. Her renal and liver functions were normal. Chest X-ray on admission showed bilateral inflammatory shadows with a cavitatory lesion on the right apical region. Chest X-ray on the third day revealed wide spread inflammatory shadows involving whole right sided lung field (Figures 1 and 2). Computed tomographic pulmonary angiogram (CTPA) was done on the third day to exclude pulmonary embolism. She had rising mycoplasma antibody titre (four fold) with normal reticulocyte count and Coombs’ test was negative. Her fasting blood sugar and lipid profile were normal. Human immune deficiency virus (HIV) and Hepatitis B and C viral infections were excluded. Antinuclear antibody (ANA) was negative. Acute DVT of the left lower limb causing total occlusion of veins below the internal iliac vein was confirmed by venous duplex. Ultrasound scan of abdomen and pelvis was normal with no lymphadinopathy. Mycobacterium tubercululosis bacillus was detected by sputum AFB (Acid fast Bacilli) staining and confirmed by culture. She was initially treated with intravenous clarythromycin for mycoplasma infection and was started on subcutaneous enoxaparin and oral warfarin for DVT. Enoxaparin was discontinued once the target International Normalizing Ratio (INR) was achieved. Initial warfarin 7 mg once a day could maintain her INR within 2–2.5. Once tuberculosis infection was confirmed, anti tuberculus treatment (ATT) was started (DOTS regimen under category- 1). With initiation of ATT, a titrated warfarin dose up to 15 mg per day was given to maintain therapeutic INR. One month after initiating warfarin, a repeat duplex scan was done and revealed little blood flow without significant recanalisation or further extension of the thrombus. Two months after the commencement of ATT, thrombophilia screening according to the National Health Services (NHS) United Kingdom Foundation Trust guidelines for thrombophilia testing was planned [5]. Protein S and C levels were normal with normal fibrinogen level. DRVVT (Dilute russell’s viper venom test) and KCT (Kaolin clotting time) were negative. Next she was discharged on warfarin 15 mg once a day. She was reviewed at the medical clinic in a week and two weeks’ time, and then she had the target INR. In subsequent clinic visits INR was in the sub therapeutic range (1.2 to 1.6). She had poor drug compliance and clinic follow up despite proper education of family members as well as the patient. She refused admission for in ward management. She defaulted clinic follow up and for about five months of initial presentation was admitted to a local hospital with sudden onset of difficulty in breathing with low blood pressure. She died a few minutes after admission. Large pulmonary embolism obstructing the left pulmonary artery was detected on post mortem examination.

## Discussion

Venous thromboembolism is a rare complication of tuberculosis. The association between inflammation, haemostatic changes and hypercoagulable state has been established in tuberculosis recently. Another mechanism of DVT is retroperitoneal adenopathies in patient**s** with tuberculosis causing compression of the inferior vena cava in the absence of any haemostatic abnormalities [6]. This is unlikely in our patient as we have excluded abdominal adenopathies by ultrasonographically.

Hypercoagulability in tuberculosis is attributed to decreased antithrombin lll and protein C, elevated plasma fibrinogen level, increased platelet aggregation and reactive thrombocytosis [7,8]. Apart from high frequency of antiphospholipid antibody levels in a patient with tuberculosis, deficiency of protein S has been mentioned [8]. But in our patient all these were normal.

Robsen *et al.* found 35 patients with pulmonary tuberculosis with DVT but only two of them, DVT was the presenting feature as in our case [9]. Some reports indicate that thrombotic phenomena in patients with pulmonary TB can occur in other sites as well [10,11].

Turken *et al.* in a case control study demonstrated haemostatic disturbances in 45 patients with active pulmonary tuberculosis [8]. It has stated that haemostatic disturbances improved within four weeks of commencing ATT [8]. For this reason, ATT should be started immediately in addition to anticoagulant treatment. So early commencement of ATT in our patient would have contributed to the absence of thrombophilic changes, as it was done after two months of treatment.

This patient who presented with lower limb DVT with pulmonary tubercolosis did not have any other risk factors or causes for the development of DVT. Thrombophilia screen for protein C, S were negative and she does not fulfill the criteria for antiphospholipid syndrome or connective tissue disorders with high risk of DVT. We could not investigate for the Factor V Leiden mutation and genetic study for prothrombin gene mutation due to its unavailability at the time of investigation and financial constraints.

Our patient was initially started on anticoagulant treatment and the target INR was achieved with a daily warfarin 7 mg dose. She was started on standard ATT according to her body weight once diagnosis of pulmonary tuberculosis was made. Then the maintenance of the target INR was difficult and a high dose of warfarin (15 mg daily) was administered to achieve the target INR. This was explained by hypercoaguability state induced by rifampicin [12] by decreasing hepatic production and increasing clearance of anticoagulants. In addition rifampicin is a cytochrome p450 inducer. Even after providing proper information on warfarin therapy, the patient’s drug compliance was poor and she defaulted in clinic follow up. All these would have contributed to her succumbing to pulmonary embolism.

Apart from DVT, our patient clinical picture was complicated with co-existent Mycoplasma infection. This was evident by initial deterioration of the chest X-ray with minimal respiratory signs and symptoms with rising Mycoplasma antibody titer.

Even though this patient was anemic it was unlikely to be due to mycoplasma induced hemolytic anemia as she had normal bilirubin levels, with normal retic count & negative Coombs test. She was from a poor socioeconomic background and had hypochromic microcytic anemia suggesting iron deficiency with menorrhagia. Iron supplement was given and her haemoglobin and blood picture was improved. Serum iron studies were not done due to financial constrains.

## Conclusion

Our case emphasizes that patients with severe pulmonary tuberculosis are at risk of developing thromboembolism due to the disease itself. Therefore clinicians should have high index of suspicion to diagnose these cases. It should be noted that even though starting ATT itself improves haemostatic disturbances, achieving target INR is difficult due to drug interactions. Thus they should be followed up closely to prevent complication and death from pulmonary embolism. At the same time clinicians should keep in mind that pulmonary tuberculosis patients can develop superadded infections as well.

## Consent

Written informed consent was obtained from the patient's husband for publication of this Case Report and any accompanying images. A copy of the written consent is available for review by the Editor-in-Chief of this journal.

## Abbreviations

DVT: Deep vein thrombosis
ESR: Erythrocyte sedimentation ratio
CRP: C reactive protein
CTPA: Computed tomographic pulmonary angiogram
HIV: Human immunodeficiency virus
ANA: Anti nuclear antibody
AFB: Acid fast Bacilli
DOT: Direct observe therapy
INR: International Normalizing ratio
DRVVT: Dilute Russell’s viper venom test
KCT: Kaolin clotting time
ATT: Anti tuberculosis treatment
TB: Tuberculosis
